# Blockade of catecholamine-induced growth by adrenergic and dopaminergic receptor antagonists in *Escherichia coli *O157:H7, *Salmonella enterica *and *Yersinia enterocolitica*

**DOI:** 10.1186/1471-2180-7-8

**Published:** 2007-01-30

**Authors:** Primrose PE Freestone, Richard D Haigh, Mark Lyte

**Affiliations:** 1 Department of Infection, Immunity and Inflammation, University of Leicester School of Medicine, Leicester, UK; 2 Department of Pharmacy Practice, School of Pharmacy, Texas Tech University Health Sciences Center, Lubbock, TX 79430, USA

## Abstract

**Background:**

The ability of catecholamines to stimulate bacterial growth was first demonstrated just over a decade ago. Little is still known however, concerning the nature of the putative bacterial adrenergic and/or dopaminergic receptor(s) to which catecholamines (norepinephrine, epinephrine and dopamine) may bind and exert their effects, or even whether the binding properties of such a receptor are similar between different species.

**Results:**

Use of specific catecholamine receptor antagonists revealed that only α, and not β, adrenergic antagonists were capable of blocking norepinephrine and epinephrine-induced growth, while antagonism of dopamine-mediated growth was achieved with the use of a dopaminergic antagonist. Both adrenergic and dopaminergic antagonists were highly specific in their mechanism of action, which did not involve blockade of catecholamine-facilitated iron-acquisition. Use of radiolabeled norepinephrine suggested that the adrenergic antagonists could be acting by inhibiting catecholamine uptake.

**Conclusion:**

The present data demonstrates that the ability of a specific pathogen to respond to a particular hormone is dependent upon the host anatomical region in which the pathogen causes disease as well as the neuroanatomical specificity to which production of the particular hormone is restricted; and that both are anatomically coincidental to each other. As such, the present report suggests that pathogens with a high degree of exclusivity to the gastrointestinal tract have evolved response systems to neuroendocrine hormones such as norepinephrine and dopamine, but not epinephrine, which are found with the enteric nervous system.

## Background

During the past decade, there has been increasing recognition that microorganisms can actively respond to the host's neurophysiological hormonal output through the utilization of neuroendocrine hormones as environmental cues to initiate growth and pathogenic processes [1,2]. The study of such microbial-neuroendocrine hormone interaction has been termed microbial endocrinology [1,2]. To date, the most studied neuroendocrine hormonal family from a microbial endocrinology perspective has been the catecholamines due to their central role in stress-mediated phenomena such as traumatic injury involving the sudden release of large amounts of catecholamines concomitant to bacterial exposure [3,4]. The catecholamines represent a group of organic compounds derived from tyrosine and consisting of a benzene ring with two adjacent hydroxyl groups and an opposing amine side chain. In metazoa, the catecholamines are responsible for a number of signalling phenomena and are generally associated with stressful events that result in high circulatory levels that prepare the organism physiologically for physical activity such as the "fight-or-flight" response.

Reports dating back over 70 years have described an association between catecholamines and microbial infectivity purportedly due to catecholamine-induced immune suppression [2]. The first mechanistic demonstration that catecholamines could directly influence bacterial growth, however, was not until 1992 when Lyte and Ernst used a serum-based medium to demonstrate that exposure to catecholamines induced log-fold increases in growth of a limited number of gram-negative pathogens [5]. The examination of a much larger set of clinical isolates by Freestone *et al *showed that recognition of catecholamines was widespread amongst Gram-positive and negative bacteria [6]. Subsequent reports have extended the range of stress hormone-responsive bacteria [7], as well as demonstrating a further role for catecholamines in the production of virulence-associated factors such as toxins [8] and adhesins [9], biofilm formation [10], and quorum sensing [11]. The question as to whether such direct microbial-catecholamine interactions occur via a receptor-mediated process has, however, remained controversial. The most likely explanation for these conflicting reports (as described below) is that the examination of a putative receptor-mediated process in bacteria has relied upon observations from mammalian systems where the identification and classification of cellular catecholamine receptors has lead to treatments for a variety of human disease conditions extending from hypertension to depression. As such, experimental approach has largely been dictated by the availability of reagents that have been developed for use in mammalian systems.

In mammals, the biochemical pathway for the synthesis of catecholamines is *L*-dopa (most commonly from food-borne sources) → Dop → NE → Epi. NE and Dop-containing sympathetic nerve terminals are distributed widely throughout the body, including the intestinal tract where they make up part of the enteric nervous system (ENS) [12]. Indeed, half of the NE present within the mammalian body is synthesized and utilized within the ENS. Epi, on the other hand, is principally produced by the adrenal glands on the kidneys and is not present within the ENS since no biosynthetic pathways have ever been found throughout the entire length of the GI tract [12]. NE and Epi bind to adrenergic-type receptors while Dop binds to dopaminergic-type receptors. The adrenergic receptors are classified into 2 major families, α and β, with a number of receptor subtypes being increasingly identified. Similarly, substantial heterogeneity of the dopamine receptor has been described, with at least 5 receptor types currently recognized [13]. Importantly, NE and Epi are able to interact and stimulate more than one adrenergic receptor family since NE can stimulate both α and β_1_, but not β_2_, adrenergic receptors. Dopamine can also interact with any of the D_1_–D_5 _receptor subtypes. While the availability of a number of highly specific antagonists has enabled the elucidation of the physiological role of the various receptor types and subtypes, this work has been almost exclusively carried out in mammalian model systems. Characterization of adrenergic receptors in non-mammalian systems utilizing pharmacological reagents developed for mammalian systems have been reported for *Tetrahymena pyriformis *[14] and *Trypanosoma cruzi *[15].

In bacteria, the initial search for a catecholamine receptor was carried out by Lyte and Ernst, who examined the ability of a range of concentrations of 3 α and β adrenergic receptor antagonists to block the ability of NE to modulate growth of several Gram-negative enteric species [16]. Since these antagonists had little effect on blocking catecholamine growth induction, it was concluded that a non-α, non-β type adrenergic receptor was present in Gram-negative bacteria. More recently, Sperandio *et. al. *[17] revisited the concept of a specific bacterial receptor for catecholamines, initially suggested by Lyte and Ernst [16], and showed that an α and β adrenergic antagonist could inhibit Epi- and NE-induced LEE gene and flagella expression in *Escherichia coli *O157:H7 [16,17]. The putative adrenergic receptors were identified as the QseBC and QseEF two component regulators that control flagella and LEE expression, respectively [11] and which were also thought to have dual specificity for the *luxS*-dependent AI-3 [17]. A more recent report [18] used *in vitro *constructs to demonstrate that NE and Epi could bind to the *E. coli *O157:H7 QseC sensor kinase and that the α adrenergic antagonist phentolamine but not the β antagonist propranolol could prevent catecholamine binding. Interestingly, the antagonist specificity of QseC appears to differ markedly between these two reports since in the prior study [17] the same research group used an *in vivo *model to report that QseC had both α and β specificity, while the latter *in vitro *study states that QseC possesses only α adrenergic specificity [18].

In order to resolve these prior conflicting reports, the present study was designed to provide a more definitive examination of the presence and nature of a putative adrenergic/dopaminergic receptor-based mechanism in bacterial-catecholamine interactions. A pharmacological approach employing a wider spectrum of adrenergic antagonists than those used in previous studies [16,17] was utilized to examine the specificity of bacterial catecholamine responsiveness in three major enteric pathogens. Additionally, antagonist to agonist ratios over a larger concentration range than previously reported in the literature for bacteria was also employed. As previous reports had not examined whether bacterial responses to dopamine were receptor-mediated, we further investigated the effects of a range of dopaminergic antagonists on dopamine-induced effects on bacterial growth.

## Results

### Antagonism of catecholamine-induced bacterial growth by adrenergic and dopaminergic receptor antagonists

Previous reports, which have utilized small numbers of eukaryotic receptor antagonists, often with limited dose-response information, have produced conflicting results regarding the existence of a putative bacterial catecholamine receptor(s) [16,17]. As shown in Table 2, we utilized an extensive range of antagonists, whose receptor specificity is shown in Table 1, which were tested over a wide dose response range. It is important to note that these bacterial pharmacological experiments were initiated with population densities of less than 10^2 ^CFU per ml in order to more closely mimic the numbers of cells likely to be involved at the initial stages of infection. The data in Table 2 demonstrate that the non-selective β-adrenergic receptor antagonist propranolol, which others had previously shown to block Epi responsiveness in *E. coli *O157:H7 [17] had no effect on the ability of NE, Epi or Dop to induce growth in any of the 3 bacterial species we tested, which included an *E. coli *O157:H7. Similar negative results were obtained with a second non-selective β-adrenergic receptor blocker labetalol. In contrast to the β-adrenergic receptor antagonists, the α-adrenergic antagonists phentolamine, phenoxybenzamine and prazosin were all able to inhibit growth induction by Epi and NE by up to three or more log-orders, in a concentration-dependent manner, as compared to control cultures not supplemented with antagonist (*P *< 0.0001) (Table 2). Examination of the kinetics of the α-adrenergic antagonist-induced blockade of growth induction by the catecholamines demonstrated that the order of addition of catecholamine and α-antagonist to the cultures was not important. However, the continuing presence of the antagonist during the culture period was required in order to observe maximal inhibition of catecholamine-induced growth, thereby indicating that the inhibition observed was not due to an irreversible binding of the α-antagonist. None of the α or β antagonists when tested alone induced growth of any of the bacterial strains tested even at concentrations as high as 500 μM. Furthermore, addition of Fe in the form of Fe(NO_3_)_3 _to catecholamine-supplemented cultures containing the α-antagonists overcame the antagonist blockade of growth induction (Table 2). This indicates that the growth inhibition by α-adrenergic receptor antagonists was not due to any cellular toxicity of the antagonist, but instead represents a specific antagonism of the bacterial response to NE and Epi. Additional experiments showed that the specific growth phase of the cultures (stationary or exponential) from which bacteria were obtained for the initial inocula in experimental cultures did not influence the potency profile of the α-antagonists. However, the initial population density of the bacterial cultures was relevant, as the cut off level of the antagonism effect in serum-SAPI medium was at approximately 10^5 ^CFU per ml for all three species (this represents the cell density at which catecholamine-independent growth occurs). Increasing the concentration of the catecholamine reduced the inhibitory effect of the adrenergic antagonists (Table 3) indicating that the inhibition observed is competitive.

Interestingly, results presented in Tables 2 and 3 also reveals that the α- or β-adrenergic antagonists tested showed very little inhibition of bacterial growth responses to Dop. From the eukaryotic receptor perspective this would not be surprising given that dopamine does not operate through either α or β-adrenergic receptors, but instead by interaction with specific dopamine receptors which are not targets for α or β-adrenergic antagonists [13]. Previous reports from our laboratories have shown that Dop modulates growth in prokaryotes through the provision of iron from the host iron binding proteins Tf and Lf in a manner similar to that shown by NE and Epi [3,19,20]. We therefore investigated whether dopaminergic antagonists had any effect on *E. coli *O157:H7, *S. enterica *and *Y. enterocolitica *growth responses to Dop. Inclusion of the non-selective antagonist haloperidol, and the D_1 _specific antagonist raclopride in Dop-supplemented serum-SAPI assays did not alter the ability of Dop to induce growth in any of the 3 bacterial species (data not shown); however, the D_2 _receptor antagonist chlorpromazine was able to block growth responses to Dop in all 3 species by up to 3 log orders (Table 4) (*P *< 0.0001). In contrast, chlorpromazine had no significant effect upon either NE or Epi induction of growth. Addition of' Fe to dopamine-supplemented *E. coli *O157:H7, *S. enterica *and *Y. enterocolitica *cultures containing chlorpromazine restored growth, again indicating that the inhibition observed was not due to toxicity of the antagonist (Table 4). Addition of chlorpromazine alone did not induce growth in any of the 3 strains tested, while increasing the concentration of the catecholamine reduced the inhibitory effect of the chlorpromazine therefore indicating that the dopaminergic antagonism observed is competitive (data not shown). The non-selective dopamine receptor agonist apomorphine did not induce growth, and was also without effect on dopamine-mediated growth induction.

### Mechanistic investigations of the adrenergic and dopaminergic antagonist inhibition of catecholamine growth responsiveness

As previously mentioned, one aspect of the mechanism by which catecholamines induce growth relates to their ability to facilitate iron removal from Tf and Lf [3,10,20,21]. Using *E. coli *strains deficient in enterobactin synthesis and uptake (*entF *and *tonB *mutations), we [19] and others [22] have shown that for Gram-negative bacteria, a functional siderophore system is an integral element in the mechanism by which bacteria assimilate the Tf/Lf-complexed iron made available by the interaction of the catecholamine with the host iron binding protein. We therefore hypothesized that a possible mechanism by which the α-adrenergic antagonists were blocking catecholamine-induced bacterial growth might be through interference with the catecholamine-mediated uptake of Fe from Tf. The ability of α-adrenergic antagonists to specifically block catecholamine-mediated uptake of Fe from Tf was examined with the use of ^55^Fe-labelled Tf as described in Materials and Methods. As shown in Table 5, bacterial ^55^Fe uptake assays were performed in the presence of NE and Epi and were challenged with concentrations of phentolamine, phenoxybenzamine or prazosin which had been shown in Table 2 to inhibit the ability of NE and Epi to induce growth by at least 2 log orders. The results of such ^55^Fe uptake assays clearly demonstrated that the α-adrenergic antagonists caused no significant reduction in the ability of the catecholamines to mediate bacterial ^55^Fe acquisition from ^55^FeTf, and that only the addition of Fe affected the amount of ^55^Fe that was internalized by each of the bacterial strains (Table 5) (due to the repression of Fe-regulated siderophore synthesis) [19,22]. The β-adrenergic antagonists propranolol and labetalol also had no effect on catecholamine-mediated ^55^Fe uptake from ^55^FeTf (data not shown). We used a similar methodology to determine whether dopaminergic antagonists affected dopamine-mediated deliver of ^55^Fe from ^55^FeTf (Table 6). Concentrations of chlorpromazine which were markedly inhibitory to dopamine stimulated growth induction also had no significant effect on uptake of ^55^Fe from ^55^FeTf with only addition of Fe causing any reduction in bacterial ^55^Fe acquisition (due again presumably to repression of siderophore synthesis) [19,22].

### Examination of the effect of α-adrenergic antagonists on norepinephrine uptake

**Figure 1:**
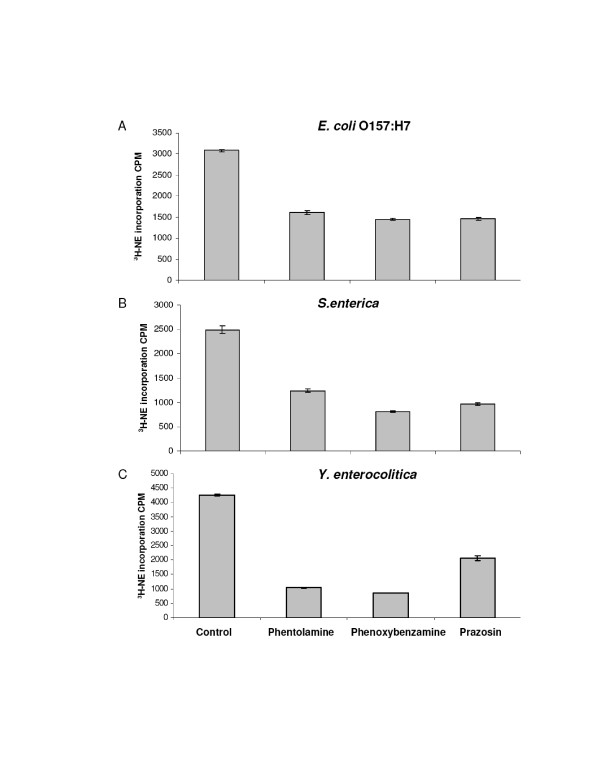
**α-Adrenergic antagonist inhibition of bacterial uptake of NE**. Washed exponential cultures of *E. coli *O157:H7, *S. enterica*, or *Y. enterocolitica *were added at a cell density of around 2 × 10^8 ^CFU/ml to DMEM containing 50 μM NE plus 5 × 10^5 ^cpm/ml of ^3^H-NE and the following: no additions (Control), or 200 μM phentolamine, phenoxybenzamine or prazosin. Cultures were incubated statically for 6 hrs at 37°C in a 5% CO_2 _humidified incubator, and analysed for growth and ^3^H-NE uptake as described in Materials and Methods. ^3^H-NE uptake assays were performed in duplicate on at least two occasions; variation within individual assay sets was 5% or less, and between experiments no more than 10%. Antagonist supplemented cultures showed statistically significant decreases in uptake of ^3^H-NE (*P *< 0.001) as compared with the Control.

Adrenergic antagonists classically exert their effects on eukaryotic cells by competitively binding to adrenergic receptors (with the exception of phenoxybenzamine, which binds irreversibly) [23]. Previously, we demonstrated that NE is internalized by bacteria during the NE-growth induction process [21], which prompted us to investigate whether the mechanism by which the adrenergic antagonists were acting might involve blocking the entry of NE into the bacterial cell. Figure 1 shows the uptake of ^3^H-NE in high cell density cultures of *E. coli *O157:H7, *S. enterica *and *Y. enterocolitica *treated with a concentration of α-adrenergic antagonist that had been shown to inhibit growth induction by NE (Table 2). The data shown reveals that for all 3 species uptake of ^3^H-NE was significantly reduced by the presence of the antagonist (*P *< 0.001), though in no case was it completely blocked.

## Discussion and Conclusion

The present report provides the most comprehensive study to date regarding the possible role of adrenergic- and dopaminergic-type receptors in catecholamine-induced bacterial growth. The results presented provide evidence for the involvement of bacterial response systems that resemble α, but not β, adrenergic receptors in the mechanism of NE and Epi growth induction of Gram-negative bacteria. Evidence was also obtained for the involvement of a bacterial response system with D_2 _dopaminergic-like specificity in dopamine-mediated growth induction.

In the vertebrate nervous system the adrenergic receptors for NE and Epi were originally classified as belonging to either α or β subtypes, but subsequent research over the last 50 years has revealed that each subtype in fact represents a receptor family that is comprised of a number of subtypes [23]. Although NE differs from Epi only by the lack of a methyl substitution in the amino group, a significant and defining physiological distinction between the two can be found in their relative ability to stimulate α and β receptors. Epi, which is principally produced in the adrenal medulla, is a potent agonist for α, β_1 _and β_2 _adrenergic receptors. NE which is produced by neurons of the sympathetic and enteric nervous systems, can stimulate both α and β_1_, but to a much lesser degree β_2_, adrenergic receptors. This differential ability to stimulate one or other adrenergic receptor subtype ultimately determines the physiological response of various tissue and organ systems both to endogenously produced catecholamines and those administered exogenously for therapeutic interventions.

Although adrenergic and dopaminergic receptors have been described in other organisms, including the malarial parasite *Trypanosoma cruzi *[15], there have been few reports examining the presence of such receptors in bacteria. An initial report by Lyte and Ernst [16] concluded that a non-α, non-β adrenergic receptor mediated process was responsible for catecholamine-induced growth of Gram-negative enteric bacteria, including *E. coli*. More recent *in vivo *work by Sperandio and co-workers [17] reported that both α and β adrenergic antagonists (phentolamine and propranolol) could block the response of *E. coli *O157:H7 to Epi and NE, suggesting that the QseC and QseE receptors have catecholamine binding sites with dual α and β adrenergic catecholamine specificity. Our present report suggests that bacterial recognition of NE and Epi in the growth context is mediated by a response system with primarily α-adrenergic specificity, since only the α-antagonists phentolamine, prazosin and phenoxybenzamine were able to block the adrenergic catecholamine growth induction process. The failure of the β-adrenergic receptor antagonists propranolol and labetalol to prevent NE and Epi induction of growth strongly suggests that the response pathway for these catecholamines is unlikely to be via either of the previously identified QseC or QseE receptors. This would also be consistent with the observation that *Y. enterocolitica *(in common with the other pathogenic *Yersinia*) does not contain homologs for QseBC or QseEF, and does not show growth responsiveness to Epi [24]. That the present results are somewhat in contradiction to the above previous adrenergic receptor reports [17,18] is not surprising, since our present study used a different and larger set of α and β receptor antagonists as well as employing a much wider range of dose-response curves for both the antagonist as well as bacterial inocula. This approach was adopted because it is well recognized that pharmacological characterization of any one adrenergic or dopaminergic receptor type or subtype requires the use of multiple antagonists, employing antagonist:agonist ratios that examine interaction over a wide dose-response range especially given that both NE and Epi exhibit multiple potencies for both α and β adrenergic receptors.

Bacterial responses to Dop, an abundant gastrointestinal catecholamine, were not addressed by either of the above receptor studies [17,18], and so we investigated whether Dop induced growth in enteric bacteria through the same signalling pathways as NE/Epi. The data in Table 4 clearly show that although a dopaminergic antagonist could almost completely block growth induction by Dop, it had no significant effect on NE or Epi responsiveness. These results indicate that, although the mechanism of dopamine-induced growth responsiveness in enteric bacteria initially appears to be similar to that of NE and Epi, there are in fact elements in the response pathway for this catecholamine that are distinct from those involved in NE or Epi signalling.

Previous studies [3,5,22,25] have shown that on a concentration-dependent basis NE is the most potent growth stimulator of all of the catecholamines in its ability to induce the bacterial species examined, inducing in serum-based media a greater than 3 log increase in cell numbers at a concentration as low as 10 μM [3,5,22,25]. For Dop or Epi, concentrations of 20 and 50 μM, respectively, were required to elicit a similar magnitude of growth stimulation [3,24]. In considering the concentration of catecholamines used in the present and past *in vitro *studies it should be emphasized that micromolar concentrations are meant to mimic the concentrations that may be present within target organs where *in vivo *experiments have shown that intra-synaptic concentrations of NE may be as high as 10^-2 ^M [26,27]. It is well appreciated that the level of catecholamines in the blood are within the nanomolar range; however, these values in large part reflect spill over from the tissues and therefore grossly underestimate local effective concentrations, particularly in the gut [27]. Interestingly, recent reports which have shown the ability of dopamine to affect the behaviour of *Caenorhabditis elegans *have utilized concentrations in the millimolar range [28,29].

The previously reported lack of growth responsiveness by *Y. enterocolitica *to Epi [5,24] is of interest as it indicates that catecholamines cannot be considered as being agents which solely mediate access to host Tf/Lf sequestered iron for bacterial growth. Further experiments from our laboratories have demonstrated that not only does Epi fail to induce growth but that it can also antagonize the growth inducing effects of both NE and Dop in *Y. enterocolitica *[24]. Although the differential action of the adrenergic and dopaminergic antagonists on *Y. enterocolitica *responses indicate that NE and Dop are likely to signal in *Y. enterocolitica *via different response pathways (as was demonstrated for *E. coli *O157:H7 and *S. enterica*) the ability of Epi to specifically antagonize *Y. enterocolitica *responsiveness to both NE and Dop [24] also suggests that the signalling pathways for these catecholamines must include common response elements.

*Y. enterocolitica *shows almost no growth responsiveness to Epi, and is almost exclusively an enteric pathogen. What does this tell us about the potential role of neuroendocrine hormones in the infectious disease process in the gut? Within the gastrointestinal tract, the 100 million neurons that comprise the ENS innervate its entire length [12]. While the presence of adrenergic and dopaminergic containing neurons has been well documented within the intestinal tract [12], as well as local production of NE and Dop [30], there have been no reports of epinephrine containing neurons. The lack of neurons with the ENS containing phenylethanolamine *N*-methyltransferase, which is needed for the synthesis of Epi from NE in the catecholamine biosynthetic pathway, is the most likely explanation why epinephrine is not found within the ENS. It has been proposed [17,18,31] that Epi is the key host-derived hormonal signal in the pathogenesis of enteric pathogens such as *E. coli *O157:H7; however if Epi were present in concentrations equivalent to those of NE and Dop, the present results [24] indicate that *Y. enterocolitica *should have significant problems in growing within the gut environment, which it clearly does not. Based upon the lack of neurophysiological and anatomical evidence for the presence of Epi in the gut, it has previously been questioned whether an infecting bacterium is likely to be exposed to significant levels of Epi while in the intestinal tract [2]. We therefore speculate that it is NE, rather than Epi, that is likely to be the cross-communicating adrenergic signal molecule between host and enteric pathogens and that the bacterial response to Epi observed in *E. coli *O157:H7 [17,18,31] is more likely related to its structural similarity to NE. Given that specific neuronal innervation in the gut has been well demonstrated for dopamine [12], our demonstration that it is possible to specifically antagonize responses to this catecholamine suggest it would be interesting to investigate whether it can also cross communicate with the Qse two-component regulator systems.

Previously, we and others [3,5-7,10,20,25,32-34] have shown that catecholamines may induce bacterial growth either through facilitating acquisition of host-sequestered Fe, which in the case of Gram-negative species requires both siderophore production and uptake systems, or through induction of a separate and distinct growth inducer. We therefore sought to determine if the mechanism of growth inhibition we observed with the adrenergic and dopaminergic antagonists involved abrogation of either or these processes. The results presented in Tables 5 and 6 showed clearly that the α-adrenergic and dopaminergic antagonists did not exert their effects either through inhibition of catecholamine-Tf complex formation and subsequent iron mobilization, nor via the siderophore-dependent processes by which Gram-negative bacteria assimilate the Tf iron release induced by the catecholamine (if siderophore production had been inhibited then the uptake of Tf iron would have been reduced, as occurred in those Table 5 and 6^55^Fe uptake assays conducted in the presence of Fe) [19,22].

Since the mode of action of the adrenergic antagonists did not appear to be via either of the mechanisms we had previously shown to be involved in NE/Epi growth induction, we examined whether they might be acting to block uptake of the catecholamine. Figure 1 showed that the α-adrenergic antagonists phentolamine, prazosin and phenoxybenzamine all reduced bacterial uptake of ^3^H-NE. Although this data points to a possible mechanism by which the adrenergic antagonists may be blocking catecholamine responsiveness (for instance, inhibition of a specific catecholamine uptake system), incorporation of ^3^H-NE into the cell was never completely blocked, suggesting for NE at least, that there may be more than a single point of entry. Indeed, the low molecular weight and structural polarity of NE does not preclude its entry into the bacterial cell by a non-specific uptake route, such as a porin, though comparison of ^3^H-NE levels in an OmpA mutant showed no difference to wildtype (data not shown). Previously, we showed that the outer membrane receptor energizer protein TonB is an essential element in the siderophore-dependent process by which *E. coli *acquires Fe from Tf-catecholamine complexes [19]. More recently, we have found evidence that TonB may also play a role in NE uptake, since cellular levels of ^3^H-NE in an *E. coli *O157:H7 TonB mutant was less than half that of its wildtype parent. Interestingly, Sperandio *et al *have also suggested that there might be an outer membrane receptor for NE, though this seems at odds with their observation that a *tonB *mutant showed normal induction of LEE in response to NE [31].

Previously, using ^3^H-NE-labelled enteropathogenic *E. coli *we demonstrated that the majority of assimilated NE is contained within the cytoplasmic/periplasmic fraction [21]. Using more precise cell fractionation methods we found that for enterohemorrhagic *E. coli *and *S. enterica *the location of internalized ^3^H-NE was again principally within the cytoplasm, with around 10–15% of the total cellular levels of ^3^H-NE present in the periplasmic space. Others have reported that that AI-3 and Epi appear to be recognized by the same outer membrane receptor, but that response to AI-3 and Epi required their transportation to the periplasm where they interact with the appropriate sensor kinase to activate gene expression [31]. Osmotic shocking the cultures in Figure 1 revealed that there was little difference in the periplasmic:cytoplasmic cellular ratio of incorporated ^3^H-NE between control and antagonist treated bacteria, indicating that there was no specific inhibition by the antagonist of the movement of ^3^H-NE through the outer membrane to the periplasm, and suggesting that the antagonists were acting to reduce total cellular uptake of NE.

If it is presumed that the mechanisms of catecholamine growth induction are largely conserved between *E. coli *O157:H7, *S. enterica *and *Y. enterocolitica*, then the combined data obtained from the experiments described in this report can be used to make predictions about the response elements that are likely to be involved in catecholamine-mediated growth induction. The differences in the specificity of adrenergic antagonist effects between our study reported herein and those of Sperandio and co-workers [31] indicates that the previously described two component regulator systems QseBC and QseEF are unlikely to be involved in the NE/Epi response pathways which lead to growth induction. We have not excluded the possibility that other as yet unidentified two component regulator systems may be involved; however, the decrease in bacterial uptake of NE in the presence of adrenergic antagonists suggests instead that their mechanism of action could be at the level of blocking uptake of catecholamine into the cell. If, as our data indicates, this blocking effect is at the level of catecholamine entry into the cytoplasm then we can infer that it is in the cytoplasm that the catecholamines then interact with a response regulator to induce growth. As we observe differential specificity in the effects of the adrenergic and dopaminergic antagonists this would necessitate two separate inner membrane uptake systems; an adrenergic uptake system for NE and Epi, and a dopaminergic uptake system for Dop. A single cytoplasmic response regulator that would recognize all three catecholamines would explain the results for *E. coli *and *S. enterica*, though in the case of *Y. enterocolitica *it would require that the homologous regulator had a different binding specificity such that it responds to NE and Dop but is blocked by the action of Epi [24]

In addition to the possibility of a cytoplasmic response regulator we have also considered that the uptake of the catecholamines could have a purely metabolic effect. As already noted, siderophore synthesis is an essential element in the Tf-dependent mechanism of catecholamine growth induction in Gram-negative bacteria [19,22]. In a serum- and blood based medium where Fe is actively withheld from the bacteria by Tf, it could be envisaged that an excess of an aromatic compound could potentially be recycled to chorismate and then channelled into the production of large amounts of enterobactin or yersiniabactin which would facilitate Fe uptake and promote growth. To investigate this possibility we have tested the ability of NE, Epi and Dop to rescue two *E. coli *mutants (*aroD *and *aroK*) that are deficient in the pathway to chorismate that is common for both enterobactin and the aromatic amino acids. None of the catecholamines could rescue the *aro *mutants in a simple growth assay using an amino acid free minimal medium indicating that, at least in *E. coli*, there is no direct pathway which could be used to recycle the catecholamine hormones to chorismate and then to enterobactin in order to promote bacterial growth.

Considered collectively, the data clearly indicates that the bacterial growth response to catecholamines involves more than the simple provision of host-derived iron that was identified in our earlier work. The evidence from the antagonist experiments indicates the presence of specific recognition systems for NE, Epi and Dop that are essential for induction of bacterial growth. Differential effects of the adrenergic antagonists indicate that these response elements are distinct from the NE- and Epi-responsive two-component regulator systems that have already been identified [18,31]; however, unequivocal evidence for the existence of bacterial α-adrenergic and dopaminergic uptake systems or receptors specific for growth induction awaits the results of further mutant analysis and microarray work, which are currently underway in our laboratories.

## Methods

### Bacterial strains and growth conditions

Recent clinical and reference isolates of *Yersinia enterocolitica *isolates were obtained from Dr. Paddy Kimmit of the Leicester Public Health Laboratory, Leicester UK. *Salmonella enterica *strain SL1344 was obtained from Dr. Jay Hinton, Institute of Food Research, Norwich, UK. *E. coli *O157:H7 strain NCTC12900 was used previously [19]. Serum-SAPI medium was prepared as described previously [5,6] and had the following composition: 6.25 mM NH_4_NO_3_, 1.84 mM KH_2_PO_4_, 3.35 mM KCl, 1.01 mM MgSO_4_and 2.77 mM glucose, pH 7.5, supplemented with 30% (v/v) adult bovine serum) (Sigma, Poole, UK). Apo-forms of human transferrin (Tf), apomorphine, chlorpromazine, haloperidol, labetalol, phenoxybenzamine, phentolamine, prazosin, propranolol, raclopride and yohombine, epinephrine, dopamine and norepinephrine were all purchased from Sigma, Poole, UK. ^55^FeCl_3 _(IES, specific activity 5 mCi/mg Fe), ^3^H-NE (TRK584, l-[7,8-^3^H] norepinephrine) were obtained from Amersham Life Science, UK. The relative specificities of the various antagonists used in this study for adrenergic and dopaminergic receptors are shown in Table 1.

### Catecholamine response and antagonism assays

Catecholamine antagonism assays were performed in serum-SAPI medium supplemented with concentrations of the compounds shown in the text. A serum-based medium was employed to more closely approximate *in vivo *conditions within a mammalian host [2]. Controls comprised equivalent volumes of the solvent used to dissolve the catecholamine or the antagonist. To determine whether an antagonist was directly inhibitory to bacterial growth, all antagonism of catecholamine-growth induction assays were also performed in the presence of a concentration of Fe which overcomes the Fe-limitation of serum-SAPI medium (100 μM Fe(NO_3_)_3_) and allows maximal bacterial growth [21]. Unless stated otherwise, bacteria were inoculated into serum-SAPI at approximately 50–100 CFU per ml. The final concentration of the bacterial inoculum was determined by standard pour-plate analysis using Luria agar. Cultures were incubated statically at 37°C in a 5% CO_2 _humidified incubator for 18 hours in the case of *E. coli *O157:H7 and *S. enterica *and for 40 hours for *Y. enterocolitica *isolates since prior reports examining growth rate of *E. coli *and *Y. enterocolitica *in serum-based medium have shown a slower growth rate for *Y. enterocolitica *as compared to *E. coli *[5]. At the end of the incubation period cultures were thoroughly re-suspended by vigorous pipetting and numbers of bacteria enumerated by standard pour plate analysis using Luria agar as previously described [21]. All growth response assays were carried out in duplicate, and all experiments were performed on at least two separate occasions. Where appropriate, statistical analysis was performed using an unpaired t-test in which a two-tailed P value was calculated (Instat program, GraphPad Software, San Diego, CA). Statistical significance was defined as a *P *value of less than 0.05.

### Analysis of effects of adrenergic and dopaminergic antagonists on catecholamine-mediated bacterial uptake of iron from transferrin (Tf)

^55^Fe-Tf was prepared as described previously by incubation of apo-Tf with ^55^FeCl_3 _using sodium citrate as the iron donor [21]. Since concentrations of antagonist were used which inhibit growth response to NE and Epi by at least 2 log orders, the use of actively metabolizing bacteria was required to examine the ability of catecholamine receptor antagonists to affect catecholamine-mediated uptake of iron from transferrin. As such, bacteria were inoculated into serum-SAPI medium at the higher cell density of 10^8 ^CFU per ml. Cultures were then supplemented with 3 × 10^5 ^counts per minute (cpm) of ^55^Fe-Tf and catecholamines and receptor antagonists added at the concentrations indicated in the figure legends. Cultures were incubated at 37°C in a 5% CO_2 _humidified incubator for 6 hrs, following which cells were harvested by centrifugation at 5000 × *g *for 5 minutes, washed in PBS and assayed for cell numbers and ^55^Fe incorporation using standard pour plate analysis and scintillation counting as described previously [21]. Assays were performed in triplicate on at least two occasions; variation within individual assay sets was less than 5%, and between experiments was usually less than 10%.

### Analysis of the effects of α-adrenergic antagonists on bacterial uptake of NE

Exponential cultures of *E. coli *O157:H7, *S. enterica*, or *Y. enterocolitica *were harvested, washed twice in DMEM medium (Sigma, Poole, UK), and added at a cell density of approximately 2 × 10^8 ^CFU per ml to 10 ml of fresh DMEM containing 50 μM NE plus 5 × 10^5 ^cpm per ml of ^3^H-NE and the following: no additions (control), or 200 μM phentolamine, phenoxybenzamine or prazosin. Cultures were incubated statically for 6 hrs at 37°C in a 5% CO_2 _humidified incubator (this incubation period was determined by conducting a prior time course of ^3^H-NE uptake and represents the time required for maximal uptake). Cells were then further analyzed for growth and ^3^H-NE uptake as described for the ^55^Fe incorporation assays above. ^3^H-NE uptake assays were performed in duplicate on at least two occasions; variation within individual assay sets was 5% or less, and between experiments no more than 15%.

## Authors' contributions

PPEF and ML conceived of the study and participated in its design and interpretation of results. PPEF and RDH performed the experiments. PPEF and ML drafted the initial manuscript with RDH contributing revisions. All the authors have read and approved the final manuscript.

## Figures and Tables

**Table 1:** Specificity of receptor antagonists and affinities.

**Antagonist**	**Specificity**
Phentolamine	α-Adrenergic (α_1_=α_2_)
Phenoxybenzamine	α-Adrenergic (α_1_=α_2_)
Prazosin	α-Adrenergic (α_1_)
Labetalol	α and β Adrenergic (α_1_,β)
Propranolol	β-Adrenergic (β_1_=β_2_)
Chlorpromazine	Dopaminergic (D_2 _> D_1_)
Haloperidol	Dopaminergic (non-selective)
Raclopride	Dopaminergic (D_1_)

**Table 2:** Antagonism of catecholamine-induced growth responsiveness

		**Antagonist Concentration (μM)**										
**Species**	**Antagonist**	**CA**	**0**	**0.1**	**1**	**10**	**20**	**50**	**75**	**100**	**200**	^b^**N/A**
***E. coli *O157:H7**	**Phentolamine**	NE	^a ^8.29	8.23	8.04	7.75	7.48	6.65	5.88	5.74	5.69	4.14
		NE+Fe	^c ^8.30								8.19	
		Epi	8.20	8.12	8.21	8.01	7.88	7.32	6.46	5.94	5.84	
		Epi+Fe	8.19								8.04	
		Dop	8.16	8.11	8.12	8.16	8.00	7.92	7.83	7.78	7.69	
												
	**Phenoxybenzamine**	NE	8.25	8.13	8.12	7.62	7.47	6.90	5.51	4.26	4.00	4.34
		NE+Fe	8.19								8.05	
		Epi	8.13	8.09	8.00	8.02	7.76	7.00	6.46	5.90	5.81	
		Epi+Fe	8.20								8.04	
		Dop	8.16	8.16	8.15	8.11	8.12	8.10	8.00	7.99	7.87	
												
	**Prazosin**	NE	8.25	8.25	8.03	8.00	7.78	7.31	6.72	5.85	5.47	4.16
		NE+Fe	8.27								8.28	
		Epi	8.12	8.16	8.13	8.08	7.94	7.81	6.43	6.12	5.66	
		Epi+Fe	8.16								8.10	
		Dop	8.13	8.12	8.11	8.09	8.12	8.10	8.08	8.04	8.00	
												
	**Propranolol**	NE	8.14	8.07	8.08	8.00	8.08	8.03	8.00	7.99	7.90	4.16
		Epi	8.13	8.11	8.14	8.10	8.21	8.12	8.13	8.08	7.88	
		Dop	8.15	8.15	8.10	8.10	8.07	8.06	8.03	8.06	8.07	
												
***S. enterica***	**Phentolamine**	NE	8.25	8.25	8.00	7.94	6.94	6.83	5.83	5.80	5.50	4.88
		Epi	8.07	8.04	8.01	7.93	7.61	6.90	5.66	5.07	4.68	
		Dop	8.09	8.07	8.05	8.12	8.12	8.04	7.99	7.82	7.34	
												
	**Phenoxybenzamine**	NE	8.27	8.23	8.20	8.14	7.90	6.62	6.41	6.34	5.18	4.89
		Epi	8.15	8.14	8.17	8.16	7.87	7.04	6.94	6.77	5.97	
		Dop	8.10	8.11	8.10	8.07	8.09	8.07	8.04	8.01	8.00	
												
	**Prazosin**	NE	8.17	8.16	8.10	8.04	7.74	7.48	6.94	5.41	5.00	4.87
		Epi	8.14	8.14	8.16	8.14	8.10	7.82	7.33	6.95	6.77	
		Dop	8.12	8.15	8.12	8.10	8.12	8.07	7.07	8.03	8.05	
												
	**Propranolol**	NE	8.15	8.14	8.10	8.07	8.19	8.16	8.06	7.98	7.75	4.89
		Epi	8.18	8.19	8.15	8.08	8.16	8.08	8.08	8.03	8.01	
		Dop	8.16	8.09	8.12	8.14	8.10	8.09	8.12	8.06	8.05	
												
***Y. enterocolitica***	**Phentolamine**	NE	8.30	8.29	8.26	7.96	7.54	6.87	6.52	5.99	5.99	3.83
		Dop	8.11	8.06	8.11	8.08	8.11	8.08	8.07	8.03	8.01	
												
	**Phenoxybenzamine**	NE	8.28	8.32	8.23	7.86	7.00	6.81	6.26	5.77	5.62	3.90
		Dop	8.09	8.07	8.11	8.10	8.08	8.07	6.10	8.03	7.99	
												
	**Prazosin**	NE	8.25	8.21	8.15	8.03	7.47	6.95	6.64	6.50	5.88	3.82
		Dop	8.05	8.07	8.08	8.07	8.08	8.06	8.04	8.01	7.94	
												
	**Propranolol**	NE	8.26	8.22	8.24	8.20	8.19	8.22	8.07	8.04	7.99	4.00
		Dop	8.05	8.08	8.09	8.11	8.12	8.07	8.08	8.03	8.04	

*E. coli *O157:H7, *S. enterica *and *Y. enterocolitica *were inoculated at approximately 10^2 ^CFU/ml into duplicate 1 mL aliquots of serum-SAPI containing the catecholamines (CA) NE, Epi and Dop plus the concentrations of antagonists shown in the table, and incubated for either 18 hours (*E. coli *and *S. enterica*) or 40 hours (*Y. enterocolitica*), and enumerated for growth (**^a^**, expressed as log^-10 ^CFU/ml) as described in Materials and Methods. Growth levels of non-catecholamine supplemented cultures (**^b^**, N/A) are shown for comparison purposes. NE and Dop were used at 50 μM, and Epi and Fe (Fe(NO_3_)_3_) at 100 μM. Note that catecholamine antagonist assays containing Fe (**^c^**) were also performed for *S. enterica *and *Y. enterocolitica *and confirmed, by no effect on bacterial growth induction by iron, that the antagonists were not toxic (data not shown). The results shown are representative data from at least three separate experiments; all data points showed variation of less than 5%. Antagonist supplemented cultures showed statistically significant decreases in growth levels (*P *< 0.001) as compared with control (catecholamine only supplemented) cultures.

**Table 3:** α-Adrenergic and dopaminergic antagonist inhibition of norepinephrine, epinephrine and dopamine-mediated growth induction is competitive

**Species**	**Catecholamine **μM	^a ^**No antagonist**	**Chlorpromazine**	**Phentolamine**	**Phenoxybenzamine**	**Prazosin**
	**NE**		N/D			
*E. coli *O157:H7	^b ^0	4.62		3.91	4.6	4.00
	5	6.77		4.64	4.66	4.77
	50	8.03		5.19	5.02	5.75
	100	8.40		5.84	6.00	6.82
	200	8.42		7.33	7.42	7.88
	500	8.41		8.24	8.33	8.24
						
	**Epi**		N/D			
	5	4.92		4.00	3.83	4.08
	50	7.28		5.19	4.32	5.72
	100	8.05		5.76	5.75	6.82
	200	8.24		7.25	7.95	7.85
	500	8.00		8.05	8.08	8.09
						
	**Dop**			N/D	N/D	N/D
	5	5.82	4.68			
	50	8.03	5.90			
	100	8.36	6.53			
	200	8.13	6.83			
	300	8.06	7.60			
						
*Y. enterocolitica*	**NE**		N/D			
	0	3.87		3.83	3.83	3.82
	5	4.65		4.00	4.32	4.47
	50	8.03		5.75	5.71	5.74
	100	8.09		6.78	5.82	6.56
	200	8.04		7.00	7.54	7.94
	500	8.00		8.02	8.05	8.00
						
	**Dop**			N/D	N/D	N/D
	5	5.66	4.62			
	50	7.47	5.19			
	100	8.11	6.12			
	200	8.02	6.50			
	300	7.78	7.95			

*E. coli *O157:H7 and *Y. enterocolitica *were inoculated at approximately 10^2 ^CFU/ml into duplicate 1 mL aliquots of serum-SAPI containing either no additions (No antagonist, 0) or only the NE, Epi and Dop concentrations shown (No antagonist). To determine if increasing the catecholamine concentration overcame antagonists effects, the same concentrations of NE or Epi or Dop were incubated with 200 μM additions of the dopaminergic antagonist chlorpromazine, or the α-antagonists phentolamine, phenoxybenzamine and prazosin. Note that usage of Dop was limited to 300 μM because higher concentrations were inhibitory to *Y. enterocolitica *(See Figure 1). Cultures were incubated for either 18 hours (*E. coli *O157:H7) or 40 hours (*Y. enterocolitica*), and enumerated for growth (**^a^**, expressed as log^-10 ^CFU/ml) as described in Materials and Methods. Growth levels of non-catecholamine supplemented cultures (0, **^b^**) are shown for comparison purposes. N/D – not determined. The results shown are representative data from two separate experiments; all data points showed variation of less than 3%. α-adrenergic antagonist-supplemented NE and Epi cultures showed statistically significant decreases in growth levels (*P *< 0.001) as compared with control (catecholamine-only supplemented) cultures. Similar results to those shown for *E. coli *O157:H7 were also obtained for *S. enterica*.

**Table 4:** Antagonism of dopamine-induced growth responsiveness

	**Chlorpromazine Concentration (μM)**											
**Species**	**CA**	**0**	**0.1**	**1**	**10**	**20**	**50**	**75**	**100**	**200**	**300**	^b^**N/A**
*E. coli *O157:H7	Dop	**^a ^**8.00	7.99	7.88	7.82	7.73	7.32	6.75	6.10	5.37	4.98	4.14
	Dop+Fe	8.12									8.16	
	NE	8.30									8.22	
	NE+Fe	8.25									8.31	
	Epi	8.06									7.88	
	Epi+Fe	8.16									8.09	
												
*S. enterica*	Dop	7.95	7.94	7.88	7.82	7.75	7.67	7.52	7.01	6.17	4.96	4.92
	Dop+Fe	8.16									8.14	
	NE	8.09									7.99	
	NE+Fe	7.99									8.20	
	Epi	8.00									7.88	
	Epi+Fe	8.21									8.16	
												
*Y.enterocolitica*	Dop	8.00	8.00	8.01	8.00	7.99	7.31	7.07	6.94	5.35	5.04	3.90
	Dop+Fe	8.05									8.03	
	NE	8.05									7.96	
	NE+Fe	8.06									8.02	
	Epi	N/D									N/D	
	Epi+Fe	N/D									N/D	

*E. coli *O157:H7, *S. enterica *and *Y. enterocolitica *were inoculated at approximately 10^2 ^CFU/ml into duplicate 1 mL aliquots of serum-SAPI containing the catecholamines (CA) and concentrations of chlorpromazine shown and incubated and enumerated for growth levels (^a^, expressed as log^-10 ^CFU/ml)as described in Materials and Methods. NE and Dop were used at 50 μM, and Epi and Fe (Fe(NO_3_)_3_) at 100 μM. The results shown are representative data from three separate experiments; data points typically showed variation of less than 3%. Growth levels of non-catecholamine supplemented cultures (^b^, N/A) are shown for comparison purposes. Chlorpromazine-supplemented cultures showed statistically significant decreases in growth levels (*P *< 0.001) as compared with control (catecholamine-only supplemented) cultures.

N/D, not determined.

**Table 5:** α-Adrenergic antagonists do not inhibit the ability of catecholamines to -mediate bacterial uptake of Tf-complexed iron.

	**Uptake of ^55 ^Fe from ^55 ^Fe-Tf (CPM)**		
**Culture Conditions**	*Y. enterocolitica*	*E. coli *O157:H7	*S. enterica*
No additions (control)	363 (14)	848 (23)	910 (33)
50 μM NE	2736 (13)	21343 (1185)	22867 (848)
50 μM NE plus 200 μM phentolamine	2375 (98)	22333 (1154)	24843 (141)
50 μM NE plus 200 μM phenoxybenzamine	2482 (93)	20205 (1058)	22106 (676)
50 μM NE plus 200 μM prazosin	3133 (37)	23189 (1185)	21109 (625)
50 μM NE plus 100 μM Fe	613 (16)	3870 (57)	6617 (246)
100 μM Epi	1759 (101)	23975 (1164)	21006 (1045)
100 μM Epi plus 200 μM phentolamine	2133 (37)	28301 (262)	21991 (827)
100 μM Epi plus 200 μM phenoxybenzamine	1987 (37)	22151 (1148)	21452 (208)
100 μM Epi plus 200 μM prazosin	1887 (104)	24682 (1059)	20403 (894)
100 μM Epi plus 100 μM Fe	238 (13)	2642 (123)	1889 (66)

The table shows NE and Epi-mediated bacterial uptake of ^55^Fe from ^55^Fe-labelled transferrin in the presence of concentrations of α-adrenergic antagonists inhibitory to NE and Epi growth induction. Exponential cultures of the bacteria shown were inoculated at approximately 2 × 10^8 ^CFU/ml into triplicate 1-ml aliquots of serum-SAPI containing the compounds shown in the Table plus 2.7 × 10^5 ^cpm of ^55^Fe-labelled transferrin. After incubation for 6 hours bacteria were harvested, washed and measured for cellular uptake of ^55^Fe from ^55^Fe-transferrin (CPM) by scintillation counting, as described in Materials and Methods. Analysis of growth levels revealed no significant differences in growth levels between control and catecholamine/antagonist supplemented cultures. The values shown represent the means of bacterial ^55^Fe incorporation from triplicate 1 ml uptake assays; standard deviations are shown in brackets.

**Table 6:** Chlorpromazine does not inhibit the ability of dopamine to mediate bacterial uptake of Tf-complexed iron.

	**Uptake of ^55 ^Fe from ^55 ^Fe-Tf (CPM)**		
**Culture Conditions**	*Y. enterocolitica*	*E. coli*	*S. enterica*
No additions (control)	125 (10)	1477 (7)	595 (72)
300 μM Chlorpromazine	231 (3)	1407 (31)	646 (11)
50 μM Dopamine	2083 (120)	24768 (750)	31877 (684)
50 μM Dopamine plus 100 μM Chlorpromazine	2239 (17)	23254 (308)	36937(217)
50 μM Dopamine plus 200 μM Chlorpromazine	2160 (152)	25945 (445)	34934 (638)
50 μM Dopamine plus 300 μM Chlorpromazine	1946 (15)	22434 (17)	33545 (131)
50 μM Dopamine plus 100 μM Fe	932 (11)	4757 (69)	3064 (77)
100 μM Fe	682 (2)	3064 (77)	2783 (28)

The table shows dopamine-mediated bacterial uptake assays of ^55^Fe from ^55^Fe-labelled transferrin in the presence of concentrations of chlorpromazine inhibitory to dopamine-mediated growth induction, and were performed as described in the legend to Table 5. The values shown represent the means of bacterial ^55^Fe incorporation from triplicate 1 ml uptake assays; standard deviations are shown in brackets. There were no significant differences in viable counts between control and test cultures.
